# An Attomolar-Level Biosensor Based on Polypyrrole and TiO_2_@Pt Nanocomposite for Electrochemical Detection of TCF3-PBX1 Oncogene in Acute Lymphoblastic Leukemia

**DOI:** 10.3390/s25175313

**Published:** 2025-08-27

**Authors:** Saulo Henrique Silva, Karen Yasmim Pereira dos Santos Avelino, Norma Lucena-Silva, Abdelhamid Errachid, Maria Danielly Lima de Oliveira, César Augusto Souza de Andrade

**Affiliations:** 1 Programa de Pós-Graduação em Inovação Terapêutica, Universidade Federal de Pernambuco, Recife 50670-901, PE, Brazil; saulohsp@gmail.com (S.H.S.); karen.avelino@ufpe.br (K.Y.P.d.S.A.); m_danielly@yahoo.com.br (M.D.L.d.O.); 2 Laboratório de Biodispositivos Nanoestruturados, Departamento de Bioquímica, Universidade Federal de Pernambuco, Recife 50670-901, PE, Brazil; 3 OX-NANO Tecnologia, Porto Digital, Recife 50030-140, PE, Brazil; 4 Instituto Aggeu Magalhães, Fundação Oswaldo Cruz (Fiocruz), Recife 50670-420, PE, Brazil; norma.silva@fiocruz.br; 5 Laboratório de Biologia Molecular, Departamento de Oncologia Pediátrica, Instituto de Medicina Integral Professor Fernando Figueira (IMIP), Recife 50070-550, PE, Brazil; 6 Institut des Sciences Analytiques (ISA), Université Claude Bernard Lyon 1, 5 rue de la Doua, 69100 Lyon, Villeurbane, France; abdelhamid.errachid-el-salhi@univ-lyon1.fr

**Keywords:** acute lymphoblastic leukemia, biosensor, cyclic voltammetry, titanium dioxide, impedance spectroscopy, polypyrrole

## Abstract

Acute lymphoblastic leukemia (ALL) represents the most common type of cancer in the pediatric population. The (1;19)(q23;p13) translocation is a primary chromosomal abnormality present in 3–12% of ALL cases. The current study aims to develop a label-free innovative nanodevice for the ultrasensitive diagnosis of the TCF3-PBX1 chimeric oncogene, featuring simplified operation and rapid analysis using minimal sample volumes, which positions it as a superior alternative for clinical diagnostics and early leukemia identification. The biosensor system was engineered on a nanostructured platform composed of polypyrrole (PPy) and a novel chemically functionalized hybrid nanocomposite of platinum nanospheres and titanium dioxide nanoparticles (TiO_2_@Pt). Single-stranded oligonucleotide sequences were chemically immobilized on the nanoengineered transducer to enable biospecific detection. Cyclic voltammetry (CV), electrochemical impedance spectroscopy (EIS), ultraviolet-visible spectroscopy (UV-Vis), and atomic force microscopy (AFM) were used to characterize each stage of the biotechnological device fabrication process. The analytical properties of the sensing tool were explored using recombinant plasmids containing the TCF3-PBX1 oncogenic sequence and clinical specimens from pediatric patients with B-cell ALL. After exposing the molecular monitoring system to the genetic target, significant variations were observed in the voltammetric oxidation current (∆I = 33.08% ± 0.28 to 124.91% ± 17.08) and in the resistance to charge transfer (ΔR_CT_ = 19.73% ± 0.96 to 83.51% ± 0.84). Data analysis revealed high reproducibility, with a relative standard deviation of 3.66%, a response range from 3.58 aM to 357.67 fM, a detection limit of 19.31 aM, and a limit of quantification of 64.39 aM. Therefore, a novel nanosensor for multiparametric electrochemical screening of the TCF3-PBX1 chimeric oncogene was described for the first time, potentially improving the quality of life for leukemic patients.

## 1. Introduction

Acute lymphoblastic leukemia (ALL) is a cancer that affects lymphoid progenitor cells. It is the most prevalent cancer in children, constituting approximately one-third of all childhood cancer cases. A genetic alteration in B-cell ALL is the TCF3-PBX1 chimeric oncogene, resulting from the chromosomal translocation t(1;19)(q23;p13) [1]. This chromosomal rearrangement fuses TCF3 (19p13) and PBX1 (1q23), producing the TCF3-PBX1 oncoprotein, which disrupts cellular differentiation [2]. Historically, this genotype is associated with poor prognosis and an increased risk of the central nervous system relapsing. However, contemporary risk-oriented treatments have improved their prognosis [3].

Genetic analysis methods have identified the TCF3-PBX1 fusion oncogene. Although karyotyping is widely used, it has limited sensitivity and is time-consuming and labor-intensive. Karyotyping requires freshly isolated neoplastic cells to be briefly cultured to obtain metaphase chromosomes [4]. The integration of fluorescent in situ hybridization (FISH) and real-time quantitative polymerase chain reaction (qPCR) into routine diagnostics overcomes some limitations of traditional methods [5,6]. FISH detects numerical cytogenetic abnormalities and locates oncogenes on chromosomes. At the same time, qPCR, the gold standard for identifying gene translocations, offers high accuracy (sensitivity 10^−4^ to 10^−5^ M) but requires prior knowledge of the fusion partner, high-quality samples, specialized personnel, and advanced equipment, and may lead to false positives [7,8]. Next-generation sequencing (NGS) has revolutionized leukemia translocation research, offering high sensitivity (~10^−6^ M), and precision in DNA and RNA sequencing [8]. Nevertheless, challenges remain, including the selection of appropriate sequencing platforms, conducting validation studies, managing procedures and interpreting results, employing bioinformatics tools, and ensuring secure data storage [9].

CV and EIS-based DNA biosensors are widely used for disease diagnosis, including the detection of cancer biomarkers, infectious diseases, and genetic disorders, due to their high sensitivity, selectivity, and ability to detect low concentrations of target analytes in complex biological samples [10]. These electrochemical biosensors and advanced genomic analysis technology emerge as promising alternatives for the molecular screening of ALL [11]. Nanotechnology-based materials, such as nanotubes, nanoparticles, nanospheres, nanocubes, nanofibers, and nanocomposites, have been employed to preserve the bioactivity of biorecognition molecules after anchoring to transductive surfaces. This approach enhances signal reproducibility, manufacturing scalability, and long-term stability [12]. The use of hybrid nanocomposites in electrochemical biosensors has attracted significant attention due to their superior performance and broad applicability, particularly those incorporating noble metals such as Au, Pt, Ag, and Pd, and metal oxides such as TiO_2_, Fe_2_O_3_, CuO, MnO_2_, and ZnO [13]. Specifically, titanium dioxide (TiO_2_) nanoparticles are a semiconductor material with energy band gaps of 3.2, 3.02, and 2.96 eV, characterized by low toxicity, high biocompatibility, resistance to photo-corrosion, photocatalytic activity, and cost-effective production. The biosensing capacity of TiO_2_ is typically attributed to its structural properties, the position of the valence band, and the generation and availability of surface holes that can accept external free electrons [14].

The association of metal nanoparticles with TiO_2_ is a strategy employed to enhance chemical stability and electrical conductivity [15]. The electronic structures of metal oxide nanomaterials can be modified through metal deposition, promoting the formation of oxygen vacancies in the interfacial region [16]. Consequently, new hybrid nanocomposites can be synthesized, such as Ag@TiO_2_/MXene [17], AuNPs/MWCNTs@TiO_2_ [18], and Ag/TiO_2_/rGO [19]. These metal–metal oxide nanocomposites may exhibit alterations in charge distribution and electronic states, thereby directly impacting electron transfer properties and biosensing efficiency [20]. Among metallic materials, platinum nanospheres (PtNs) exhibit a substantial surface area and adsorption capacity, facilitating biomolecule binding and electrochemical signal transduction [21].

Recent advances in nanomaterial design, such as the one-step microwave synthesis of PPy/zinc-based nanohybrids, enhance electron transfer properties for ultrasensitive DNA detection, achieving detection limits as low as 7.73 × 10^−21^ M [22]. These results highlight the synergistic effects between the intrinsic conductivity of PPy and the physicochemical properties of metal oxide nanoparticles, reinforcing their relevance in biosensing platforms [13,14]. PPy attributes that contribute to the enhancement of analytical robustness in biological detection include high conductivity, rapid electron transfer kinetics at the electrode/solution interface, charge storage capacity, redox reversibility, relative biological compatibility, and the presence of chemical groups for the targeted anchoring of biomolecules [23]. Compared to other conductive polymers such as poly (3,4-ethylenedioxythiophene):polystyrenesulfonic acid (PEDOT:PSS) and polyaniline (PANI), PPy exhibits superior electrochemical stability under acidic conditions, commonly used in electropolymerization [24]. Although PEDOT:PSS offers high electrical conductivity, its aqueous dispersion can compromise structural integrity during the assembly of multilayer biosensors [25]. Furthermore, PANI presents limited redox activity at neutral pH, restricting its applicability in physiological environments [26].

In this investigation, a DNA electrochemical biosensor was introduced for the first time to specifically detect the TCF3-PBX1 chimeric oncogene. Notably, the developed biosensing system incorporates a novel composite material (TiO_2_@Pt), which, in conjunction with a PPy film, facilitated the achievement of exceptionally low detection limit, on the order of attomolar. Moreover, the system demonstrates reproducibility, high selectivity, analytical accuracy, cost-effectiveness, and fast response time. Its performance was validated using both synthetic DNA samples (plasmid DNA) and clinical specimens from leukemia patients, highlighting its potential as a promising tool for laboratory diagnosis in cases of acute lymphoblastic leukemia (ALL).

## 2. Materials and Methods

### 2.1. Reagents

Pyrrole (98%), citrate-functionalized PtNs (30 nm diameter), titanium dioxide nanoparticles (TiO_2_, <100 nm), 3-aminopropyltriethoxysilane (APTES, ≥98%), 3-mercaptopropyltrimethoxysilane (MPTMS, 95%), ethanol (≥99.5%), ammonia, hydrochloric acid (HCl) (37%), glacial acetic acid (C_2_H_4_O_2_, ≥99.7%), glutaraldehyde (25% solution), bovine serum albumin (BSA, ≥98%), potassium ferricyanide (K_3_[Fe(CN)_6_], ≥99.0%), potassium ferrocyanide (K_4_[Fe(CN)_6_], 98.5–102.0%), sodium phosphate monobasic and dibasic (≥99.0%), and 0.05 μm alumina paste (α-Al_2_O_3_) were obtained from Sigma Aldrich Co. (St. Louis, MO, USA). Trizol was acquired from Invitrogen Co. Ltd. (Carlsbad, CA, USA). Analytical-grade solutions were prepared using ultrapure water from a Milli-Q Plus purification system (Billerica, MA, USA).

### 2.2. Oligonucleotide Probes and Real Samples

The clinical and plasmid samples were assessed using conventional PCR and agarose gel electrophoresis in the presence of ethidium bromide. Subsequently, the chimeric DNA sequences were cloned into the pTA vector. The analytical results of the biosensor were validated against a standard diagnostic method. Patients diagnosed with leukemia underwent iliac bone aspiration, and clinical samples were collected with their informed consent from the Pediatric Oncology Service biorepository at the Institute of Integral Medicine, Prof. Fernando Figueira (IMIP, Recife, PE, Brazil). Genetic analysis was performed on bone marrow samples after total RNA extraction from 5 × 10^6^ cells using Trizol reagent. Reverse transcription was conducted to obtain cDNA using an oligonucleotide primer. The oligonucleotide sequences of primers and probes used in the molecular biology assays are shown in Table S1. This study was approved by the Research Ethics Committee of the Aggeu Magalhães Institute (FIOCRUZ Pernambuco) with the Certificate of Presentation for Ethical Appreciation (CAAE) no. 40388420.5.0000.5190.

### 2.3. Synthesis of the TiO_2_@Pt Nanocomposite

Ethanol (40 mL) was used to disperse 100 mg of TiO_2_. Subsequently, 200 μL of APTES and 200 μL of MPTMS were added to the suspension, followed by the gradual addition of a water–ammonia solution (1:1, *v*/*v*). After continuous stirring for 10 h, the TiO_2_ nanoparticles were functionalized with terminal amino groups. The functionalized particles were collected by extensive centrifugation and washing and finally dispersed in 10 mL of water. Next, 0.5 mL of the previously modified TiO_2_ was mixed with 1 mL of PtNs (0.05 mg/mL). The mixture was sonicated for 30 min and then left for 12 h at room temperature. The TiO_2_@Pt hybrid nanocomposite was obtained by centrifuging the mixture, which was dissolved in 5 mL of water to form a gray and stable suspension [27].

### 2.4. Conception of the Biosensing Platform

**Figure 1:**
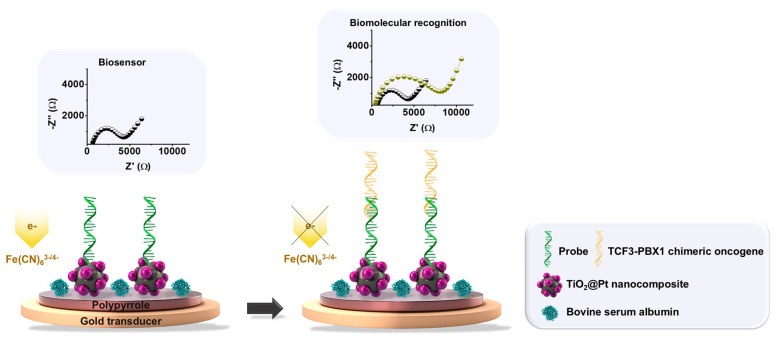
Illustration depicting the conceptual stages involved in assembling the electrochemical biosensor. A gold electrode was modified with an electropolymerized polypyrrole (PPy) film, chemically conjugated to the TiO_2_@Pt hybrid nanocomposite. Biofunctionalization of the PPy-TiO_2_@Pt platform was achieved through covalent immobilization of single-stranded DNA probes specific to the TCF3-PBX1 oncogene. Nonspecific binding sites on the sensor surface were blocked with bovine serum albumin (BSA). Upon exposure to a sample containing the TCF3-PBX1 oncogene, the PPy-TiO_2_@Pt-Probe-BSA biosensor facilitated hybridization with target DNA sequences, resulting in an enhanced impedimetric response. The representative Nyquist plots illustrate the biosensor performance and biomolecular recognition process.

Initially, the gold electrode (diameter = 2 mm) was polished with an alumina suspension (Al_2_O_3_) containing particles of 0.05 μm. Subsequently, it was rinsed with ultrapure water, subjected to ultrasonic agitation for 10 min to eliminate residual particles, and air-dried. The first stage of the sensing platform involved the electrochemical polymerization of PPy using 20 mL of a 0.5 M HCl solution containing 30 mM pyrrole monomer. The PPy used in this work was doped with chloride ions during its electropolymerization to enhance electrical conductivity. Five voltammetric cycles were conducted in the potential range of −0.4 to +1.0 V at a scan rate of 100 mV/s. The second stage involved the chemical conjugation of the TiO_2_@Pt hybrid nanocomposite, previously modified with APTES, onto the polymeric film. Initially, a crosslinking agent, glutaraldehyde (2 μL), was used to bind the amino groups of PPy and the TiO_2_@Pt nanocomposite through the formation of Schiff bases. Next, a 2 μL colloidal solution was added for 25 min to obtain a self-assembled nanostructured layer. The third stage consisted of biofunctionalizing the PPy-TiO_2_@Pt interface platform with DNA probes for the identification of the TCF3-PBX1 fusion oncogene. The immobilization of oligonucleotide sequences was achieved by adding 2 μL of 0.5% glutaraldehyde, followed by a 10-min incubation. Subsequently, 2 μL of DNA solution at a concentration of 25 pmol/μL was applied for biomolecular immobilization over 20 min. Finally, the fourth stage involved blocking the nonspecific sites of the sensor layer with BSA protein. In this step, 2 μL of a 1% BSA solution at pH 7.4 was incorporated onto the electrode surface to create the PPy-TiO_2_@Pt-Probe-BSA system (Figure 1).

### 2.5. Studies of Genetic Detection

The sensitivity and specificity of the electrochemical biosensor were evaluated through hybridization studies with recombinant plasmids containing the TCF3-PBX1 chimeric oncogene at concentrations ranging from 3.58 aM to 357.67 fM. Additionally, clinical samples (cDNA specimens) obtained from three distinct patients with acute lymphoblastic leukemia (ALL) carrying different chromosomal translocations, t(1;19), t(4;11), and t(17;19), were used for genetic screening. Although all three translocations are associated with ALL, only the t(1;19) subtype expresses the TCF3-PBX1 fusion gene and was therefore employed as a positive control. In contrast, the t(4;11) and t(17;19) samples, which do not contain the target fusion, were used as negative controls to assess the biosensor’s selectivity. The biosensor was exposed to 2 μL of the sample for 15 min to facilitate the biorecognition process. Before this step, the genetic material was thermally denatured at 94 °C for 5 min to promote the separation of double-stranded DNA into single strands, thereby enhancing the accessibility of the target sequences for specific hybridization with the immobilized DNA probe. All biological samples were diluted in phosphate-buffered saline (PBS, 10 mM, pH 7.4) and stored frozen at −22 °C. Prior to analysis, the samples were thawed and maintained under refrigeration at 4 °C only during the experimental procedures.

### 2.6. Electrochemical Measurements

Electroanalytical measurements were performed using an Autolab PGSTAT 128 N in potentiostatic mode (Metrohm Autolab Inc., Netherlands), controlled by NOVA 1.11 software. A conventional three-electrode configuration with an electrochemical cell was employed, with the electrodes immersed in a supporting electrolyte of PBS (10 mM, pH 7.4) containing 10 mM K_4_[Fe(CN)_6_]/K_3_[Fe(CN)_6_] (1:1, *v*/*v*). The working electrode was a biomodified gold electrode (ϕ = 2 mm), while Ag/AgCl (saturated with 3 M KCl) and a platinum wire served as the reference and counter electrodes, respectively. Cyclic voltammograms were obtained by applying potentials between −0.2 and +0.7 V at a scan rate of 50 mV/s to characterize the interfacial properties. Cole–Cole diagrams were recorded over a frequency range of 100 mHz to 100 kHz with an amplitude of 10 mV and an integration time of 0.125 s. The electrochemical analysis was performed independently at least three times at room temperature within a Faraday cage.

### 2.7. AFM and UV-Vis Spectroscopic Analyses

The atomic force microscope (SPM-9500; Shimadzu Corporation, Tokyo, Japan) was employed for the morphological characterization of the biosensor and its interactions with the TCF3-PBX1 oncogene. Non-contact mode was used in AFM with cantilevers equipped with an aluminum-coated silicon probe (Nanoworld, Misato, Japan; resonant frequency = 300 kHz; force constant = 42 N/m) at room temperature. Topographic micrographs (512 points per line) were acquired over a scan area of 5 × 5 µm. The resulting 3D images were processed and analyzed using Gwyddion software (version 2.59). Absorbance spectra were recorded in the range of 200 to 700 nm using a UV-VIS spectrophotometer (model K37-UVVIS from KASVI, São José dos Pinhais, Brazil).

## 3. Results and Discussion

### 3.1. Topographic Analysis

**Figure 2:**
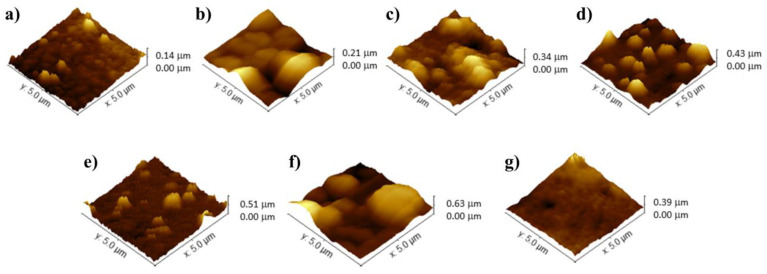
AFM images of the following films: PPy (**a**), PPy-TiO_2_@Pt (**b**), PPy-TiO_2_@Pt-Probe (**c**), PPy-TiO_2_@Pt-Probe-BSA (**d**), Biosensor with ALL positive plasmid sample (**e**), Biosensor with ALL positive clinical sample (**f**), and Biosensor with ALL negative sample (**g**).

AFM was employed to monitor the surface characteristics of the biosensor and to investigate biomolecular interactions involving the TCF3-PBX1 chimeric oncogene in biological samples (Figure 2). During the microscopic analyses, fundamental parameters such as average roughness (Ra), root-mean-squared roughness (Rq), and the maximum height of surface irregularities were evaluated. The potentiodynamic synthesis of PPy resulted in a polymer matrix with a nodular morphology, characterized by an average thickness of 140 ± 5.04 nm (Ra = 14.48 nm; Rq = 18.66 nm) (Figure 2a). According to the literature, electropolymerized PPy thin films exhibit a cauliflower-like surface topography, which enhances electrolyte permeation and electrical conductivity [28]. The development of such structures depends on various factors, including the oxidation–reduction states of the polymer, concentration, properties of the doping ions, thickness of the polymer film, and the specific electrosynthesis parameters applied [29].

Following the chemical conjugation of the hybrid nanocomposite, a PPy-TiO_2_@Pt film was fabricated, exhibiting prominent spherical structures with a peak height of up to 210 ± 8.82 nm (Ra = 21.42 nm; Rq = 28.83 nm) (Figure 2b). The nanomaterial was uniformly distributed across the PPy layer, indicating a homogeneous deposition of the TiO_2_@Pt composite. This uniformity results from the surface functionalization of TiO_2_ nanoparticles with APTES, which introduces terminal amine groups that enhance chemical affinity. These groups enable strong interfacial interactions with the nitrogen-rich polypyrrole matrix, leading to stable anchoring and uniform dispersion of the nanocomposite on the polymer surface [30]. The significant increase in roughness and surface heterogeneity, coupled with an increase in the maximum height to 340 ± 12.98 nm (Ra = 58.91 nm; Rq = 75.65 nm) after the covalent immobilization of DNA probes, highlights the effectiveness of the functionalization process. This results in a substantial alteration in the film’s topography, suggesting a robust integration of the oligonucleotide sequences onto the nanostructured transducer, as depicted in Figure 2c. The electrode modification, achieved by adding BSA molecules to block non-functional sites on the biosensor, was evidenced by the presence of well-distributed and discernible peaks and troughs, reaching a roughness of 430 ± 16.77 nm (Ra = 72.99 nm; Rq = 87.80 nm) (Figure 2d). Exposure of the PPy-TiO_2_@Pt-Probe-BSA biosensor to the TCF3-PBX1 oncogene resulted in a notable increase in surface roughness: approximately 80 nm for the plasmid sample and 200 nm for the clinical cDNA sample, with respective maximum heights of 510 ± 20.91 nm (Ra = 87.30 nm; Rq = 122.40 nm) and 630 ± 27.72 nm (Ra = 121.50 nm; Rq = 168.30 nm) (Figure 2e,f). The selectivity assay with non-complementary genetic sequences exhibited a non-granular morphology with maximum peaks at 390 ± 13.26 nm (Ra = 77.13 nm; Rq = 83.40 nm) (Figure 2g). These results suggest the sensing activity of the bioanalysis system for detecting genomic diseases.

### 3.2. UV-Vis Characterization

The UV-Vis spectroscopy analysis of TiO_2_ nanoparticles revealed a surface plasmon resonance band, as depicted in Figure S1. The absorption spectrum of TiO_2_ nanoparticles exhibited a distinct absorption edge located around 350 nm, attributed to the band gap absorption characteristics of the material [31]. In contrast, platinum nanospheres exhibited negligible absorbance in the visible region, consistent with their limited optical activity [32]. The TiO_2_@Pt hybrid nanocomposite, obtained via surface modification of TiO_2_ with APTES and MPTMS followed by conjugation with Pt nanospheres, exhibited a significant shift in the absorption edge to approximately 400 nm, along with a broadened absorption profile. These spectral changes confirm the successful formation of the hybrid nanostructure and suggest strong interfacial electronic interactions between TiO_2_ and Pt, as evidenced by the peak broadening and absorption intensity.

### 3.3. Electrochemical Measurements of the Biosensing Platform

#### 3.3.1. Voltammetry Experiments

**Figure 3:**
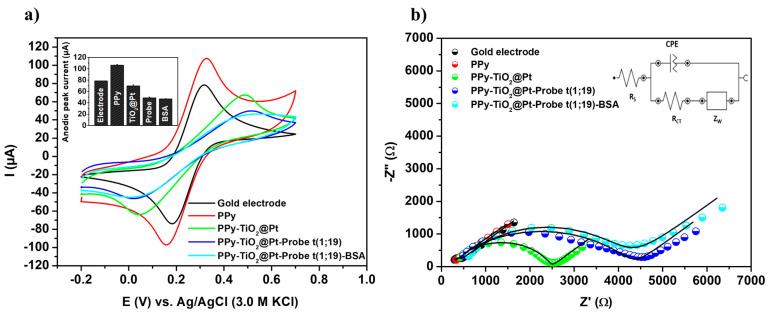
Cyclic voltammograms (**a**) and impedance diagrams (**b**) obtained from the characterization of the nanostructured biosensor fabrication steps, incorporating PPy, TiO_2_@Pt, Probe t(1;19), and BSA. Inset: Anodic peak currents recorded during biosensor development and the equivalent circuit used for fitting the spectroscopic measurements. Three consecutive analyses were performed for each methodological procedure, with experimental values reported as the mean ± standard deviation.

In Figure 3a, the bare gold electrode exhibited symmetric and well-defined anodic and cathodic peaks, characterized by an anodic peak current (Ipa) of 78.36 ± 0.16 μA and a cathodic peak current (Ipc) of −76.24 ± 0.33 μA, respectively. These findings indicate that the oxidation and reduction reactions of the electrolytic components on the unmodified gold electrode were governed by diffusion-limited transport kinetics. The electropolymerization of the pyrrole monomer led to an enhancement in the voltammetric signal, with an increase in oxidation (Ipa = 106.30 ± 1.11 μA) and reduction (Ipc = −97.25 ± 2.75 μA) currents due to PPy-mediated electron transfer. This study demonstrates that the presence of the PPy polymer can significantly improve the charge transfer efficiency of bioanalytical devices, thereby enhancing their chemical stability and sensitivity [33]. Furthermore, PPy enhances selectivity by providing an electroactive and chemically stable matrix that enables the oriented and stable immobilization of specific biomolecules, while simultaneously minimizing nonspecific adsorption [23].

Following the molecular structuring of the TiO_2_@Pt nanocomposite on the polymeric matrix, a reduction in the voltammetric area (Ipa = 69.58 ± 1.98 μA and Ipc = −64.45 ± 0.60 μA) was observed. The integration of the hybrid nanomaterial into the PPy polymer resulted in a substantial increase in surface area, the introduction of amino groups (-NH_2_) for covalent immobilization of oligonucleotides, and the establishment of a biocompatible microenvironment, which is critical for preserving the conformation of immobilized biomolecules. A further reduction in voltammetric response (Ipa = 48.75 ± 1.26 μA; Ipc = −45.94 ± 0.43 μA) was noted after the chemical immobilization of DNA probes onto the PPy-TiO_2_@Pt nanostructured transducer. This phenomenon is attributed to the reduced accessibility of the redox probe within the sensor system, primarily due to electrostatic repulsion between the phosphate groups of the DNA probes and the negatively charged [Fe(CN)_6_]^3−^/[Fe(CN)_6_]^4−^ ions. Consequently, this leads to a partial hindrance of oxidation–reduction reactions in regions close to the surface of the biomodified electrode. BSA molecules are employed to block nonspecific binding sites on the biosensor, resulting in a further decrease in voltammetric currents (Ipa = 46.90 ± 0.45 μA; Ipc = −46.12 ± 1.19 μA).

The electrochemically active surface areas of the electrodes were calculated using the Randles-Sevcik equation [34]:

(1) $$Ip=2.69\times 10^{5}n^{3/2}AD^{1/2}Cv^{1/2}$$

where Ip is the anodic peak current (A), n is the number of electrons involved in the redox reaction (n = 1 for [Fe(CN)_6_]^3−/4−^), A is the electrode surface area (cm^2^), C is the concentration of the redox species (10^−5^ mol cm^−3^), D is the diffusion coefficient of ferricyanide (6.67 × 10^−6^ cm^2^ s^−1^), and v is the scan rate (0.05 V s^−1^). The calculated surface areas were 0.0504 cm^2^ for the unmodified gold electrode, 0.0684 cm^2^ for the PPy-modified electrode, 0.0448 cm^2^ for the PPy-TiO_2_@Pt-modified electrode, 0.0314 cm^2^ for the PPy-TiO_2_@Pt-Probe electrode, and 0.0302 cm^2^ for the PPy-TiO_2_@Pt-Probe-BSA electrode. The increased surface area of the PPy-modified electrode, compared to the unmodified gold electrode, results from enhanced roughness and porosity, facilitating improved electrolyte interaction and electron transfer. The progressive decrease in surface area following the addition of TiO_2_@Pt, the DNA probe, and BSA is attributed to partial surface coverage and electrostatic repulsion of the redox probe.

#### 3.3.2. Impedance Examinations

To support the voltammetric investigation of the DNA biosensor, EIS analyses were conducted (Figure 3b). The impedance responses were rigorously analyzed by fitting them to a theoretical simulation of the experimental data using Randle’s equivalent circuit (inset of Figure 3b). The values of the circuit components, as determined from the impedance fitting analysis, are presented in the Supplementary Information (Table S3). The impedance spectrum exhibited an almost linear response, characterized by a low charge transfer resistance (R_CT_ = 0.45 ± 0.004 kΩ) for the bare gold electrode. The reduction in electrochemical resistance from 0.45 ± 0.004 kΩ (gold electrode) to 0.38 ± 0.001 kΩ (PPy-modified gold electrode) confirmed the formation of a conductive film on the metal surface. This decrease in resistance indicates successful modification and enhanced conductivity due to the PPy layer. Furthermore, an increase in the charge transfer resistance of the electrical double layer (R_CT_ = 2.28 ± 0.04 kΩ) was observed after the chemisorption of the hybrid nanocomposite. This electrochemical alteration suggests effective interaction and immobilization of the TiO_2_@Pt nanomaterial on the electrode surface. Moreover, the observed behavior can be attributed to the inherently insulating nature of the TiO_2_-based composite, combined with steric hindrance imposed by its structural architecture, which together compromise electron transfer dynamics at the electrochemical interface [21]. The covalent anchoring of bioreceptor molecules on the PPy-TiO_2_@Pt platform resulted in an increased surface resistance (R_CT_ = 4.19 ± 0.07 kΩ). Lastly, the addition of BSA molecules further increased the electrochemical impedance, resulting in a higher charge transfer resistance (R_CT_ = 4.21 ± 0.01 kΩ). The addition of BSA resulted in an elevated R_CT_, indicating effective surface blocking that minimizes nonspecific adsorption and enhances biosensor selectivity.

### 3.4. Optimization of Experimental Variables

#### 3.4.1. Electrodeposition of PPy Films

Figure S2 presents the voltammetric data obtained during the potentiodynamic polymerization of pyrrole. Over 15 cycles of polymerization, the electrochemical characteristics of the electrode surface were investigated, revealing a reduction in anodic current after the 5th scanning cycle. This observation is consistent with previous studies and can be attributed to an increase in the resistance of the conducting polymer layer, a decrease in electrical double-layer capacitance, and a reduction in the availability of pyrrole monomers during synthesis [35]. Consequently, to achieve a reproducible polymeric coating with maximal conductivity and controllable thickness, five electropolymerization cycles were determined to be optimal.

In this context, the electrochemical synthesis of PPy provides precise control over the polymerization process, allowing for the regulation of polymer thickness, morphology, and conductivity, resulting in well-defined and highly conductive films. Moreover, electrochemical synthesis is a green and cost-effective approach, minimizing the use of hazardous reagents and reducing waste generation. The simplicity and efficiency of this method contribute to the scalability and applicability of PPy-based biosensors in various biotechnological domains.

#### 3.4.2. Study of Chemical Conjugation of the TiO_2_@Pt Nanocomposite

The investigation examined varying adsorption times (10, 15, 20, 25, and 30 min) for the TiO_2_@Pt hybrid composite on the PPy-modified gold electrode. The voltammetric curves became increasingly sigmoidal with longer adsorption times, showing a decrease in both anodic and cathodic currents (Figure S3a). As illustrated in Figure S3b, the diameter of the Cole–Cole semicircle expanded proportionally with the adsorption time. These results indicate that the incubation duration affects the quantity of nanomaterial deposited on the transducer, leading to a corresponding alteration in the resistance signal. However, R_CT_ analysis (inset of Figure S3b) revealed that a 25-min incubation period results in electrode saturation, with a moderate inhibition of redox reactions occurring within the electrical double layer. Thus, the 25-min time point was identified as optimal for the adsorption kinetics and molecular arrangement of the TiO_2_@Pt nanocomposite on the polymeric film.

The ratio of PPy to TiO_2_@Pt directly influences the analytical sensitivity of the biosensor by modulating the available surface area for biomolecular interactions and the electron transfer properties of the transducer. The 25-min adsorption time corresponds to a composition that maximizes the interface between the conductive polymer and the nanocomposite, balancing electron mobility with the probe immobilization capacity. More extended adsorption periods may oversaturate the electrode surface, impairing signal transduction efficiency.

#### 3.4.3. Chemical Conjugation of the Oligonucleotide Sequences

The anchoring time for oligonucleotide sequences onto the PPy-TiO_2_@Pt nanostructured transducer was evaluated at intervals of 10, 15, 20, 25, and 30 min, showing that the most efficient adsorption occurred during the 20-min incubation period (Figure S3c,d). A gradual decrease in redox peaks and an increase in R_CT_ values (R_CT_ ranged from 2.56 ± 0.11 kΩ to 4.19 ± 0.07 kΩ) were observed between 10 and 20 min. However, prolonged incubation times did not result in significant changes in electrochemical responses, suggesting a relatively constant density of immobilized DNA probes.

### 3.5. Analytical Performance of the Sensing Platform

**Figure 4:**
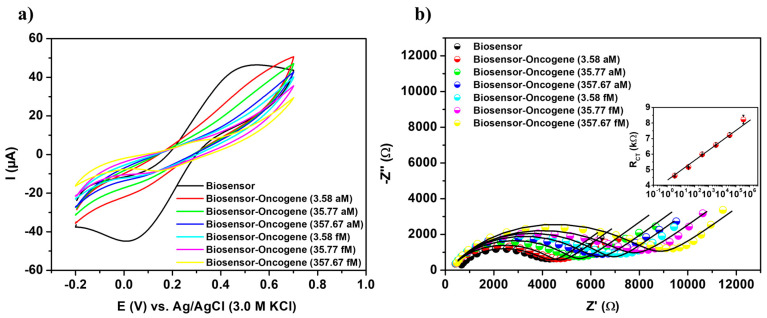
Cyclic voltammograms (**a**) and impedance diagrams (**b**) of the biosensor exposed to various concentrations of the TCF3-PBX1 chimeric oncogene in recombinant plasmids. Inset: calibration plot of the bioanalytical system with the semilogarithmic linear regression equation given by y = 4.31587 + 0.64008⋅ln(x), R^2^ value of 0.99752, SD of 4.12156, and *p*-value < 0.0001. Three consecutive analyses were performed for each methodological procedure, with experimental values reported as the mean ± standard deviation.

The analytical performance of the biosensor was evaluated against a range of recombinant plasmid concentrations containing the TCF3-PBX1 chimeric oncogene, from 3.58 aM to 357.67 fM (Figure 4). A reduction in the voltammetric signal was observed, characterized by a decrease in both anodic and cathodic peak heights, after exposure of the PPy-TiO_2_@Pt-Probe-BSA system to synthetic DNA samples (Figure 4a). The reduction in current values is proportional to the increasing DNA concentration at the transducer interface, resulting from a biomolecular barrier to electron transport. This electrochemical phenomenon reflects the biochemical recognition process, wherein the hybridization of DNA strands leads to increased electrostatic repulsion between [Fe(CN)_6_]^3−/4−^ ions and the negatively charged phosphate groups of the oncogene molecules.

The mathematical expression for the relative deviation of the anodic current variation (ΔI) is provided below:

(2) $$\Delta I\left(\%\right)=\;\frac{I_{b}-I_{a}}{I_{b}}\;\times 100$$

I_b_ and I_a_ represent the anodic peak currents before and after the oncogenic capture, respectively. The ∆I values increased linearly with the concentration of synthetic DNA samples. Consequently, a maximum variation of 58.27% was observed following the biodetection assay conducted on a plasmid sample at a concentration of 357.67 fM (Table S2).

The results of the sensitivity assay, as depicted in the Cole–Cole diagram (Z’/Ω × Z”/Ω), offer a comprehensive visual representation of changes in the system’s electrical impedance in response to varying concentrations of the TCF3-PBX1 chimeric oncogene (Figure 4b). A gradual correlation between R_CT_ values and bioanalytical responses was observed over the range of sample concentrations tested. A progressive increase in R_CT_ values (from 4.56 ± 0.11 kΩ to 8.22 ± 0.37 kΩ) was noted as synthetic DNA concentrations increased (Table S3). The substantial increase in R_CT_ values along with the concentration gradient highlights the biosensor’s capacity to detect and respond to variations in analyte levels.

R_CT_ was used to generate the analytical calibration graph (inset Figure 4b). The semilogarithmic linear fit equation, y = 4.31587 + 0.64008⋅ln(x), exhibited a high coefficient of determination (R^2^) of 0.99752. In this equation, A = 4.31587 corresponds to the intercept, B = 0.64008 represents the slope, y denotes the R_CT_ (kΩ), and x represents the analyte concentration (aM), with ln(x) being used in the regression model. The biosensing platform demonstrated a measurement range from 3.58 aM to 357.67 fM. LOD, LOQ and sensitivity were first estimated in the signal domain (kΩ), according to LOD = (3 × σ)/B, LOQ = (10 × σ)/B and sensitivity = B/electrode area, where σ corresponds to the standard deviation of triplicate blank measurements. These signal thresholds were subsequently converted into concentration values (aM) by inverting the semilogarithmic calibration equation, yielding

(3) $$x_{LOD}=\exp\frac{LOD-A\;}{B}$$

(4) $$x_{LOQ}=\exp\frac{LOQ-A\;}{B}$$

Through this statistically consistent two-step procedure, the assay achieved a LOD of 19.31 aM, a LOQ of 64.39 aM, and a sensitivity of 20.37 kΩ·aM^−1^·cm^−2^. This approach harmonizes dimensional consistency with analytical reliability, thereby providing an accurate representation of the sensor’s true performance [36,37,38,39].

A relative standard deviation of 3.66% indicates that the genosensor exhibited satisfactory reproducibility. Reproducibility was assessed by calculating the percentage value of the standard deviation from three distinct biosensors, all fabricated using identical methodological protocols and experimental conditions on the same day. Repeatability was confirmed by a relative standard deviation of less than 4.54% across all tests involving varying concentrations of the oncogene. Repeatability was evaluated by subjecting each sensor device to a minimum of three tests within a single day, assessing its electrochemical performance. The stability of the biosensing platform (PPy-TiO_2_@Pt-Probe-BSA) was evaluated over a 7-day period under optimal storage conditions (4–5 °C). The impedimetric response (R_CT_) was recorded daily, yielding an average resistance of 4.36 ± 0.11 kΩ with a relative standard deviation of 2.52%. These results indicate that the biosensor maintains consistent performance over time, suggesting potential for extended operational stability.

To further elucidate the analytical capability of the developed biosensor, Table 1 presents a comparative assessment of its performance metrics against other electrochemical detection technologies documented in the literature for leukemia genetic biomarkers, highlighting its competitive sensitivity and detection limits.

### 3.6. Electrochemical Screening in Biological Samples

**Figure 5:**
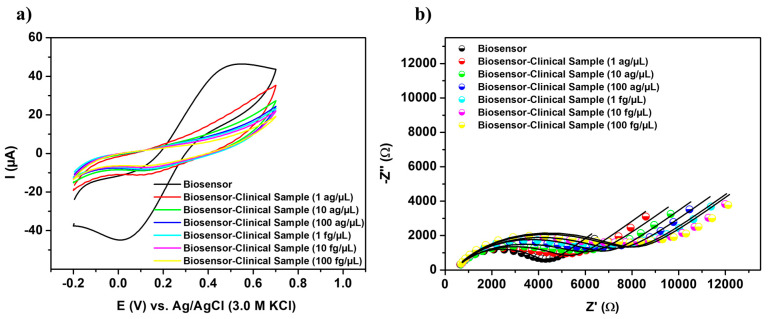
Cyclic voltammograms (**a**) and impedance diagrams (**b**) obtained from the biosensor upon exposure to clinical samples from patients diagnosed with ALL, with concentrations ranging from 1 ag/µL to 100 fg/µL.

Biomolecular detection tests were conducted using cDNA specimens obtained from patients diagnosed with ALL to assess the robustness of genetic measurements. Reduced current responses, as illustrated in Figure 5a, were observed after the biosensor interacted with biological samples. These positive samples, which express the TCF3-PBX1 chimeric oncogene at concentrations ranging from 1 ag/µL to 100 fg/µL, induced significant signal variations and sigmoid voltammograms, with ΔI values ranging from 33.08% ± 0.28 to 124.91% ± 17.08, respectively (Table S2). Discrepancies in the amplitudes of electroanalytical signals can be attributed to variations in the expression levels of the chromosomal translocation t(1;19)(q23;p13) and, consequently, to the progression of blood cell cancer. These findings substantiate the viability and efficacy of the nanotechnological apparatus for gene screening in clinical cDNA samples.

Figure 5b shows that capturing oncogenic sequences on the biosensor surface leads to a gradual enlargement of the Nyquist semicircles, which correlates with the molecular concentration of the target. This behavior indicates a reduction in charge flux within the electrical double layer, attributed to the insulating properties of hybridized DNA structures. Given the excellent suitability of the R_CT_ component for monitoring the biosensing phenomenon, the biosensor’s analytical performance was characterized by the relative variation of the R_CT_ (ΔR_CT_) as follows:

(5) $$\Delta R_{CT}=\;\frac{R_{CT\left(Biosensor-oncogene\right)}-\;R_{CT\left(Biosensor\right)}}{R_{CT\left(Biosensor\right)}}\;\times 100$$

The term R_CT(Biosensor-oncogene)_ denotes the value measured after the recognition of the TCF3-PBX1 chimeric oncogene, while R_CT(Biosensor)_ represents the initial response of the PPy-TiO_2_@Pt-Probe-BSA biosensor (Table S3). Specimens that tested positive for ALL exhibited a significant change in impedance, as indicated by the relative change in ΔR_CT_, which ranged from 19.73% ± 0.96 to 83.51% ± 0.84.

The degree of surface coverage (θ) was used as an additional parameter for diagnostic assessment. The determination of θ values following the interaction of the biodetection platform with clinical cDNA samples was performed using the following equation:

(6) $$\theta \;\left(\%\right)=1-\;\frac{R_{CT\left(Biosensor\right)}}{R_{CT\left(Biosensor-oncogene\right)}}\;\times 100$$

R_CT(Biosensor)_ represents the R_CT_ value of the PPy-TiO_2_@Pt-Probe-BSA biosensor. In contrast, R_CT(Biosensor-oncogene)_ denotes the corresponding value for the biosensor when exposed to clinical samples. Surface coverage is directly related to the amount of oncogenic DNA hybridized with an oligonucleotide probe on the analytical platform. The presence of hybridized nucleic acids on the nanostructured sensor layer leads to an increase in surface coverage. Distinct θ values were computed for various concentrations of patient specimens, ranging from 1 ag/µL to 100 fg/µL. The obtained values ranged from 16.48% ± 0.67 to 45.51% ± 0.25, corresponding to the respective concentrations of the samples. Based on the information presented in Figure S4, which shows a logarithmic curve with a tendency towards saturation, it is suggested that concentrations above 100 fg/µL may lead to the saturation of binding sites on the biosensor surface. In this context, the biosensor’s ability to conduct assays for identifying target DNA in clinical samples was demonstrated.

### 3.7. Biological Interferent Studies

**Figure 6:**
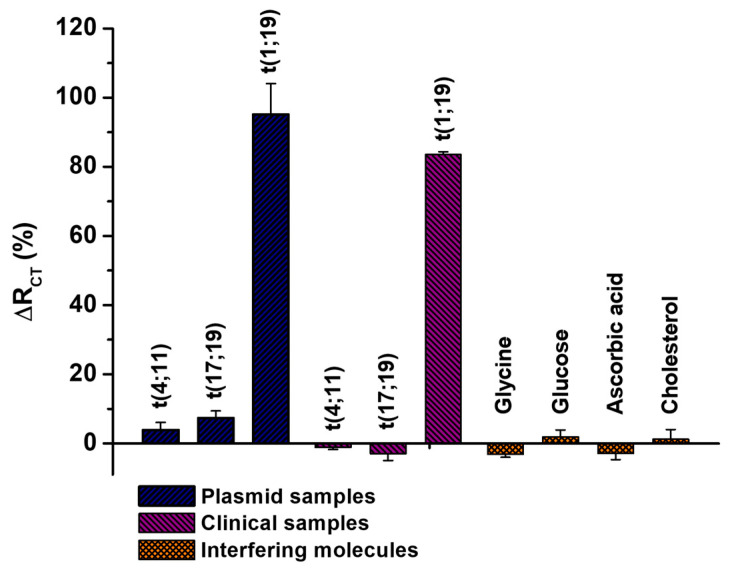
Percentage variation of R_CT_ for the biosensing platform when exposed to recombinant plasmid samples and clinical specimens containing the t(4;11), t(17;19), or t(1;19) translocations. Additionally, assays were conducted with biological interferers, such as glycine, glucose, ascorbic acid, and cholesterol. Three consecutive analyses were performed for each methodological procedure, with experimental values reported as the mean ± standard deviation.

In the present study, we investigated the applicability of the electrochemical biodevice in a specificity assay, utilizing recombinant plasmid samples and clinical specimens containing non-specific chromosomal translocations, such as t(4;11) and t(17;19) (Figure 6). It is important to note that these clinical specimens are cDNA samples containing, in addition to the t(4;11) and t(17;19) translocations, the patient’s constitutive genetic material, which may also interfere with the bioanalytical results. The results revealed that the biosensor exhibited non-significant ΔR_CT_ (%) values, ranging from −2.96% ± 1.94 to 7.39% ± 1.99, as detailed in Table S3. This range of values indicates the biosensor’s ability to distinguish samples in the absence of the target analyte effectively.

Furthermore, non-significant ΔR_CT_ values were observed when the biosensing system was exposed to samples containing common blood interferents, such as glucose, glycine, cholesterol, and ascorbic acid, even at concentrations up to 100 mg/dL (Table S3) [45]. These findings suggest that biological interferents do not compromise the functionality of the biosensor, thereby improving the method’s reliability for practical applications. In contrast, we observed a pronounced sensitivity of the biomolecular system when exposed to recombinant plasmid samples (ΔR_CT_ = 95.14% ± 8.89) and clinical specimens containing the t(1;19) chromosomal translocation (ΔR_CT_ = 83.51% ± 0.84). These results emphasize the biosensor’s capacity to specifically detect the t(1;19) chromosomal translocation. Finally, the dynamic response curves illustrating the biosensor’s analytical performance for the target oncogene, as well as its specificity against non-complementary sequences and biomolecular interferents, are presented in Figure S5 (Supplementary Material).

From this perspective, the integration of the TiO_2_@Pt nanocomposite into an electroconductive PPy film enabled the development of the first nanostructured biosensor documented in the scientific literature for detecting the TCF3-PBX1 chimeric oncogene. The proposed bioanalytical instrumentation requires minimal volumes of biological specimens, specifically two microliters, facilitating the rapid and precise recognition of the (1;19)(q23;p13) chromosomal translocation. To the best of our knowledge, this bioanalytical tool has achieved the lowest detection limit for leukemic translocations, reaching the attomolar scale. Additionally, it exhibits a notable quantification limit, as well as high sensitivity and specificity, which are critical parameters for the early assessment of malignant disorders and the monitoring of minimal residual disease.

## 4. Conclusions

The developed electrochemical system stands as a potential method for the precise investigation of the TCF3-PBX1 chimeric oncogene, a significant biomarker in ALL. The innovative nanotechnology platform, incorporating a PPy electrosynthesized layer and the TiO_2_@Pt functional nanocomposite, exhibits enhanced surface characteristics, improved electron transport, and a biologically compatible microenvironment. Demonstrating exceptional analytical robustness, sensitivity, specificity, and selectivity, the biosensing tool proved effective in biodetection tests utilizing minimal volumes of recombinant plasmid samples and clinical specimens. The biosensor demonstrates label-free electroanalytical signals measured at regular intervals, with limits of detection and quantification in the attomolar range. The high specificity of the sensor system makes it a promising tool for delivering negative test results for samples lacking the target analyte, thereby minimizing false positives and enhancing test reliability. On the other hand, the biosensor’s remarkable sensitivity in detecting the t(1;19) chromosomal translocation suggests its potential as an effective screening tool for identifying patients with this specific condition. To our knowledge, this biosensor represents the first nanostructured biodevice specifically designed for the investigation of the TCF3-PBX1 chimeric oncogene. Therefore, the nanotechnological apparatus can be considered a promising tool for clinical applications in the early diagnosis of ALL and the monitoring of minimal residual disease, potentially impacting the quality of life for cancer patients.

## Figures and Tables

**Table 1:** A comparative analysis of the biosensor developed in this study relative to other biological detection technologies documented in the literature for the electrochemical investigation of leukemia genetic biomarkers.

Sensing Strategy	Molecular Target	Analytical Technique	Hybridization Marker	Detection Time	Detection Range	Limit of Detection	Limit of Quantification	Sensitivity	Reference
Gold transducer/PPy/TiO_2_@Pt/Probe/BSA	TCF3-PBX1 chimeric oncogene	CV and EIS	Label-free	15 min	3.58 aM to 357.67 fM	19.31 aM	64.39 aM	20.37 kΩ/aM cm^2^	This work
Indium Tin Oxide/PPy/Graphene quantum dots/Probe_APLB_ Indium Tin Oxide/PPy/Graphene quantum dots/Probe_M7_	PML/RARα fusion gene	CV and EIS	Label-free	15 min	1 pM to 100 pM	0.214 pM for APLB sequence 0.677 pM for M7 sequence	0.648 pM for APLB sequence 2.05 pM for M7 sequence	--	[40]
Gold transducer/PPy/Nanocomposite of chitosan and zinc oxide nanoparticles/Probe/BSA	BCR/ABL fusion gene	CV and EIS	Label-free	15 min	138.80 aM to 13.88 pM	1.34 fM	4.08 fM	34.03 μA/fM cm^2^	[41]
Glassy carbon electrode/Gold nanoparticles/Hemoglobin-capped gold nanoclusters stabilized graphene nanosheets/Probe	BCR/ABL fusion gene	CV, EIS and differential pulse voltammetry (DPV)	Methylene blue	30 min	0.1 aM to 10 pM	0.03 fM	--	--	[42]
Magnetic glass carbon electrode/Enzyme-linked DNA magnetic beads	BCR/ABLp210 fusion gene	Amperometry	Horseradish peroxidase	--	Two linear relationships: 1 aM to 50 fM 1 fM to 1 pM	1 aM	--	--	[43]
Glassy carbon electrode/Reduced graphene oxide/Gold nanoparticles	MicroRNA-128	CV, EIS and square wave voltammetry (SWV)	Methylene blue	40 min	0.01 fM to 0.09 fM	0.00956 fM	--	--	[44]

## Data Availability

Data will be made available on request.

## Supplementary Materials

The following are available online at https://www.mdpi.com/article/10.3390/s25175313/s1, Figure S1: UV-Vis absorption spectra for TiO_2_ nanoparticles, Pt nanospheres and TiO_2_@Pt hybrid nanocomposite; Figure S2: Potentiodynamic electrochemical profiles in PPy polymerization; Figure S3: Investigation of optimal experimental parameters. Cyclic voltammograms (a) and impedance diagrams (b) of the TiO_2_@Pt composite film formed at various adsorption times. Cyclic voltammograms (c) and impedance diagrams (d) of the chemical immobilization of the DNA probe on the nanostructured platform at different adsorption intervals. The adsorption times investigated were 10, 15, 20, 25, and 30 min. Inset: Histograms depicting the R_CT_ values. Three consecutive analyses were performed for each methodological procedure, with experimental values reported as the mean ± standard deviation; Figure S4: Surface coverage degree as a function of different concentrations of specimens from patients with ALL. Three consecutive analyses were performed for each methodological procedure, with experimental values reported as the mean ± standard deviation; Figure S5: Dynamic response curves showing the biosensor’s response to varying concentrations of the TCF3-PBX1 chimeric oncogene (t(1;19)) in recombinant plasmid samples and clinical samples from patients with ALL, ranging from 1 ag/µL to 100 fg/µL (a). Dynamic response curves illustrating the biosensor’s response to clinical samples with t(4;11) and t(17;19) translocations, as well as biological interferents (glycine, glucose, ascorbic acid, and cholesterol) at 100 mg/dL (b). Three consecutive analyses were performed for each methodological procedure, with experimental values reported as the mean ± standard deviation; Table S1: Oligonucleotide sequences of primers and probes employed in the molecular biology assays; Table S2: Anodic and cathodic peak currents during the construction steps of the DNA biosensor after exposure to analytical samples. Three consecutive analyses were conducted for each methodological procedure, and the experimental values are reported as the mean values ± standard deviation; Table S3: The values of the equivalent circuit elements were obtained by fitting the impedance results for each step of biodevice assembly. Three consecutive analyses were conducted for each methodological procedure, and the experimental values are reported as the mean values ± standard deviation.
