# Supplementary material for: An Attomolar-Level Biosensor Based on Polypyrrole and TiO2@Pt Nanocomposite for Electrochemical Detection of TCF3-PBX1 Oncogene in Acute Lymphoblastic Leukemia
Source: Sensors (Basel). 2025 Aug 27;25(17):5313. doi: 10.3390/s25175313 (PMC12431060; doi:10.3390/s25175313)
Supplement: Supplementary file 1 [file sensors-25-05313-s001.zip › sensors-3779000-supplementary.pdf]

Electronic Supporting Material

AN ATTOMOLAR-LEVEL BIOSENSOR BASED ON POLYPYRROLE AND TIO_2@PT NANOCOMPOSITE FOR ELECTROCHEMICAL DETECTION OF TCF3-PBX1 ONCOGENE IN ACUTE LYMPHOBLASTIC LEUKEMIA

Saulo H. Silva[a, b, c], *Karen Y. P. S. Avelino*[a, b, c], *Norma Lucena-Silva*[d, e], *Abdelhamid Errachid*[f*], *Maria D. L. Oliveira*[a, b, c], *Cesar A. S. Andrade*[a, b, c]

[a] Programa de Pós-Graduação em Inovação Terapêutica, Universidade Federal de Pernambuco, 50670-901 Recife, PE, Brazil.

[b] Laboratório de Biodispositivos Nanoestruturados, Departamento de Bioquímica, Universidade Federal de Pernambuco, 50670-901 Recife, PE, Brazil.

[c] OX-NANO Tecnologia, Porto Digital, 50030.140 Recife, PE, Brazil.

[d] Instituto Aggeu Magalhães, Fundação Oswaldo Cruz (Fiocruz), 50670-420 Recife, PE, Brazil.

[e] Laboratório de Biologia Molecular, Departamento de Oncologia Pediátrica, Instituto de Medicina Integral Professor Fernando Figueira (IMIP), 50070-550 Recife, PE, Brazil.

[f] Université Claude Bernard Lyon 1, Institut des Sciences Analytiques (ISA), 5 rue de la Doua, 69100, Lyon, Villeurbane, France

*To whom correspondence should be addressed.

Abdelhamid Errachid, Université de Lyon, Institut des Sciences Analytiques, UMR5280, CNRS, Villeurbanne, France.

E-mail: abdelhamid.errachid-el-salhi@univ-lyon1.fr

Materials and methods

Reagents

Pyrrole (98%), citrate-functionalized PtNs (30 nm diameter), titanium dioxide nanoparticles (TiO_2, <100 nm), 3-aminopropyltriethoxysilane (APTES, ≥98%), 3-mercaptopropyltrimethoxysilane (MPTMS, 95%), ethanol (≥99.5%), ammonia, hydrochloric acid (HCl) (37%), glacial acetic acid ($C_2H_4O_2$, ≥99.7%), glutaraldehyde (25% solution), bovine serum albumin (BSA, ≥98%), potassium ferricyanide ($K_3[Fe(CN)_6]$, ≥99.0%), potassium ferrocyanide ($K_4[Fe(CN)_6]$, 98.5–102.0%), sodium phosphate monobasic and dibasic (≥99.0%), and 0.05 µm alumina paste (α-Al_2O_3) were obtained from Sigma Aldrich Co. (St. Louis, USA). Trizol was acquired from Invitrogen Co. Ltd. (Carlsbad, CA, USA). Analytical-grade solutions were prepared using ultrapure water from a Milli-Q Plus purification system (Billerica, USA).

Oligonucleotide probes and real samples

The clinical and plasmid samples were assessed using conventional PCR and agarose gel electrophoresis in the presence of ethidium bromide. Subsequently, the chimeric DNA sequences were cloned into the pTA vector. The analytical results of the biosensor were validated against a standard diagnostic method. Patients diagnosed with leukemia underwent iliac bone aspiration, and clinical samples were collected with their informed consent from the Pediatric Oncology Service biorepository at the Institute of Integral Medicine, Prof. Fernando Figueira (IMIP, Recife, PE, Brazil). Genetic analysis was performed on bone marrow samples after total RNA extraction from 5×10^6 cells using Trizol reagent. Reverse transcription was conducted to obtain cDNA using an oligonucleotide primer. The oligonucleotide sequences of primers and probes used in the

molecular biology assays are shown in Table S1. This study was approved by the Research Ethics Committee of the Aggeu Magalhães Institute (FIOCRUZ Pernambuco) with the Certificate of Presentation for Ethical Appreciation (CAAE) no. 13296913.3.0000.5190.

Synthesis of the TiO_2@Pt nanocomposite

Ethanol (40 mL) was used to disperse 100 mg of TiO_2. Subsequently, 200 μL of APTES and 200 μL of MPTMS were added to the suspension, followed by the gradual addition of a water-ammonia solution (1:1, v/v). After continuous stirring for 10 hours, the TiO_2 nanoparticles were functionalized with terminal amino groups. The functionalized particles were collected by extensive centrifugation and washing, and finally dispersed in 10 mL of water. Next, 0.5 mL of the previously modified TiO_2 was mixed with 1 mL of PtNs (0.05 mg/mL). The mixture was sonicated for 30 minutes and then left for 12 hours at room temperature. The TiO_2@Pt hybrid nanocomposite was obtained by centrifuging the mixture, which was dissolved in 5 mL of water to form a gray and stable suspension [1].

Conception of the biosensing platform

Initially, the gold electrode was polished with an alumina suspension (Al_2O_3) containing particles of 0.05 μm. Subsequently, it was rinsed with ultrapure water, subjected to ultrasonic agitation for 10 minutes to eliminate residual particles, and air-dried. The first stage of the sensing platform involved the electrochemical polymerization of PPy using 20 mL of a 0.5 M HCl solution containing 30 mM pyrrole monomer. Five voltammetric cycles were conducted in the potential range of -0.4 to +1.0 V at a scan rate

of 100 mV/s. The second stage involved the chemical conjugation of the TiO_2@Pt hybrid nanocomposite onto the polymeric film. Initially, a crosslinking agent, glutaraldehyde (2 µL), was used to bind the amino groups of PPy and the TiO_2@Pt nanocomposite through the formation of Schiff bases. Next, a 2 µL colloidal solution was added for 25 minutes to obtain a self-assembled nanostructured layer. The third stage consisted of biofunctionalizing the PPy-TiO_2@Pt interface platform with DNA probes for the identification of the TCF3-PBX1 fusion oncogene. The immobilization of oligonucleotide sequences was achieved by adding 2 µL of 0.5% glutaraldehyde, followed by a 10-minute incubation. Subsequently, 2 µL of DNA solution at a concentration of 25 pmol/µL was applied for biomolecular immobilization over 20 minutes. Finally, the fourth stage involved blocking the nonspecific sites of the sensor layer with BSA protein. In this step, 2 µL of a 1% BSA solution at pH 7.4 was incorporated onto the electrode surface to create the PPy-TiO_2@Pt-Probe-BSA system.

Studies of genetic detection

The sensitivity and specificity of the electrochemical biosensor were evaluated through hybridization studies with recombinant plasmids containing the TCF3-PBX1 chimeric oncogene at concentrations ranging from 3.58 aM to 357.67 fM. Additionally, a clinical sample (cDNA specimen) obtained from patients with t(1;19) translocation-positive acute lymphoblastic leukemia was utilized for genetic screening. The biosensor was exposed to 2 µL of the sample for 15 minutes to facilitate the biorecognition process. Prior to the biorecognition assay, the genetic material was denatured at 94°C. All biological samples were diluted in phosphate-buffered saline (PBS, 10 mM, pH 7.4) and stored frozen.

Electrochemical measurements

Electroanalytical measurements were performed using an Autolab PGSTAT 128 N in potentiostatic mode (Metrohm Autolab Inc., Netherlands), controlled by NOVA 1.11 software. A conventional three-electrode configuration with an electrochemical cell was employed, with the electrodes immersed in a supporting electrolyte of PBS (10 mM, pH 7.4) containing 10 mM $K_4[Fe(CN)_6]/K_3[Fe(CN)_6]$ (1:1, v/v). The working electrode was a nanobiomodified gold electrode (ϕ = 2 mm), while Ag/AgCl (saturated with 3 M KCl) and a platinum wire served as the reference and counter electrodes, respectively. Cyclic voltammograms were obtained by applying potentials between -0.2 and +0.7 V at a scan rate of 50 mV/s to characterize the interfacial properties. Cole-Cole diagrams were recorded over a frequency range of 100 mHz to 100 kHz with an amplitude of 10 mV and an integration time of 0.125 s. The electrochemical analysis was performed independently at least three times at room temperature within a Faraday cage.

AFM and UV-Vis spectroscopic analyses

The atomic force microscope (SPM-9500; Shimadzu Corporation, Tokyo, Japan) was employed for the morphological characterization of the biosensor and its interactions with the TCF3-PBX1 oncogene. Non-contact mode was used in AFM with cantilevers equipped with an aluminum-coated silicon probe (Nanoworld, Japan; resonant frequency = 300 kHz; force constant = 42 N/m) at room temperature. Topographic micrographs (512 points per line) were acquired over a scan area of 5 × 5 µm. The resulting 3D images were processed and analyzed using Gwyddion software. Absorbance spectra were recorded in the range of 200 to 700 nm using a UV-VIS spectrophotometer (KASHI, model K37-UVVIS).

Figure S1. AFM images of the following films: PPy (a), PPy-TiO_2@Pt (b), PPy-TiO_2@Pt-Probe (c), PPy-TiO_2@Pt-Probe-BSA (d), Biosensor with ALL positive plasmid sample (e), Biosensor with ALL positive clinical sample (f), and Biosensor with ALL negative sample (g).

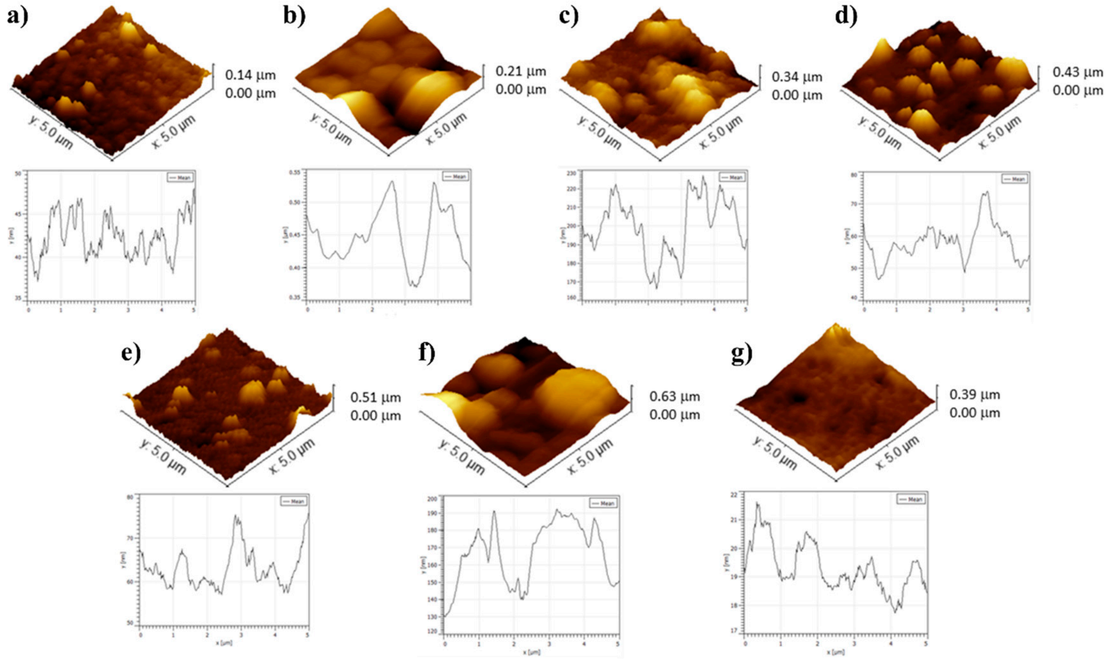

Figure S2. UV-Vis absorption spectra for TiO_2 nanoparticles, Pt nanospheres and TiO_2@Pt hybrid nanocomposite.

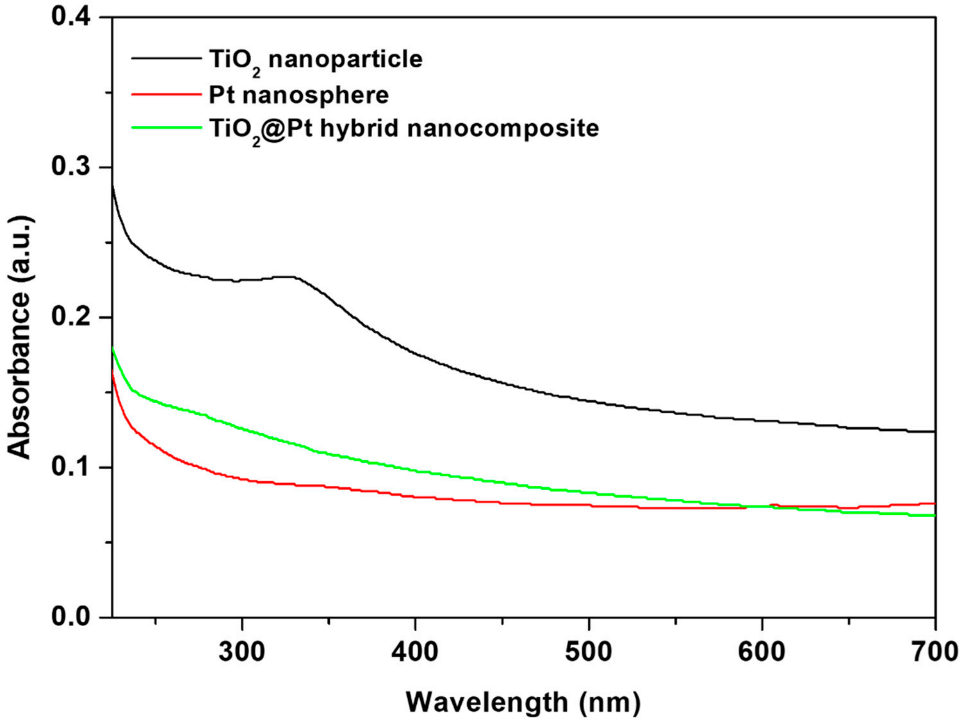

Figure S3. Potentiodynamic electrochemical profiles in PPy polymerization.

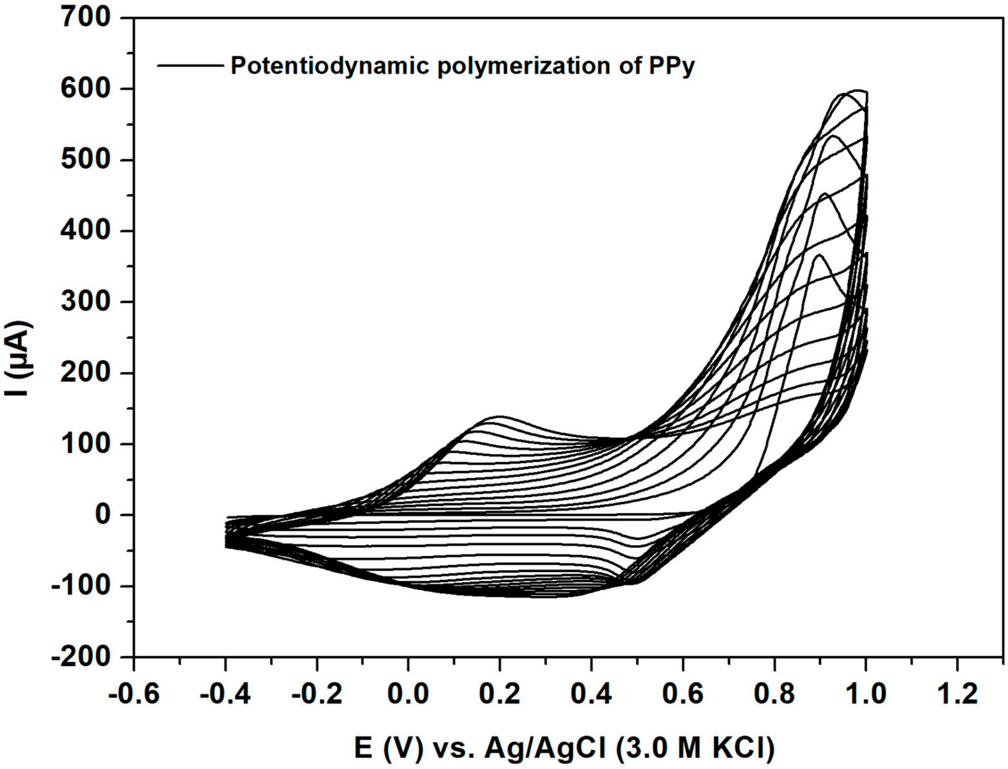

Figure S4. Investigation of optimal experimental parameters. Cyclic voltammograms (a) and impedance diagrams (b) of the TiO_2@Pt composite film formed at various adsorption times. Cyclic voltammograms (c) and impedance diagrams (d) of the chemical immobilization of the DNA probe on the nanostructured platform at different adsorption intervals. The adsorption times investigated were 10, 15, 20, 25, and 30 minutes. Inset: Histograms depicting the R_{CT} values. Three consecutive analyses were performed for each methodological procedure, with experimental values reported as the mean ± standard deviation.

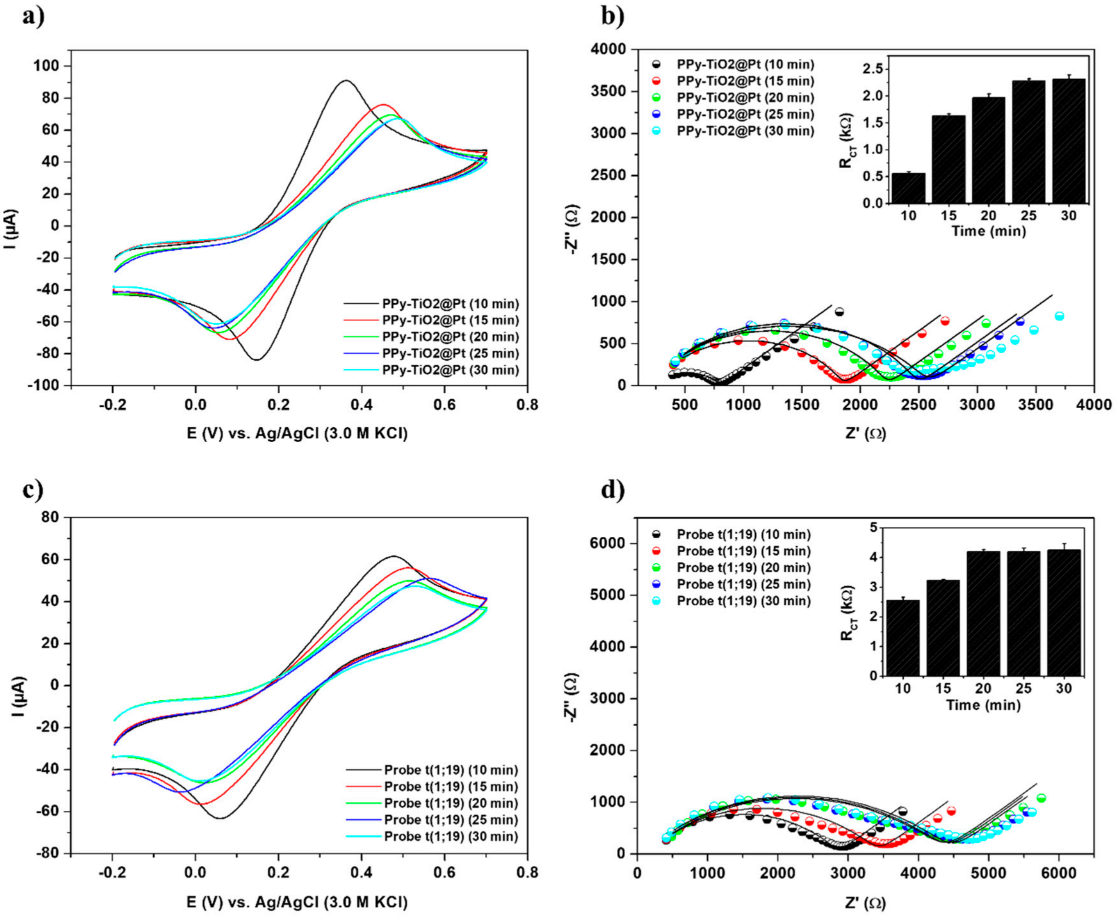

Figure S5. Surface coverage degree as a function of different concentrations of specimens from patients with ALL.

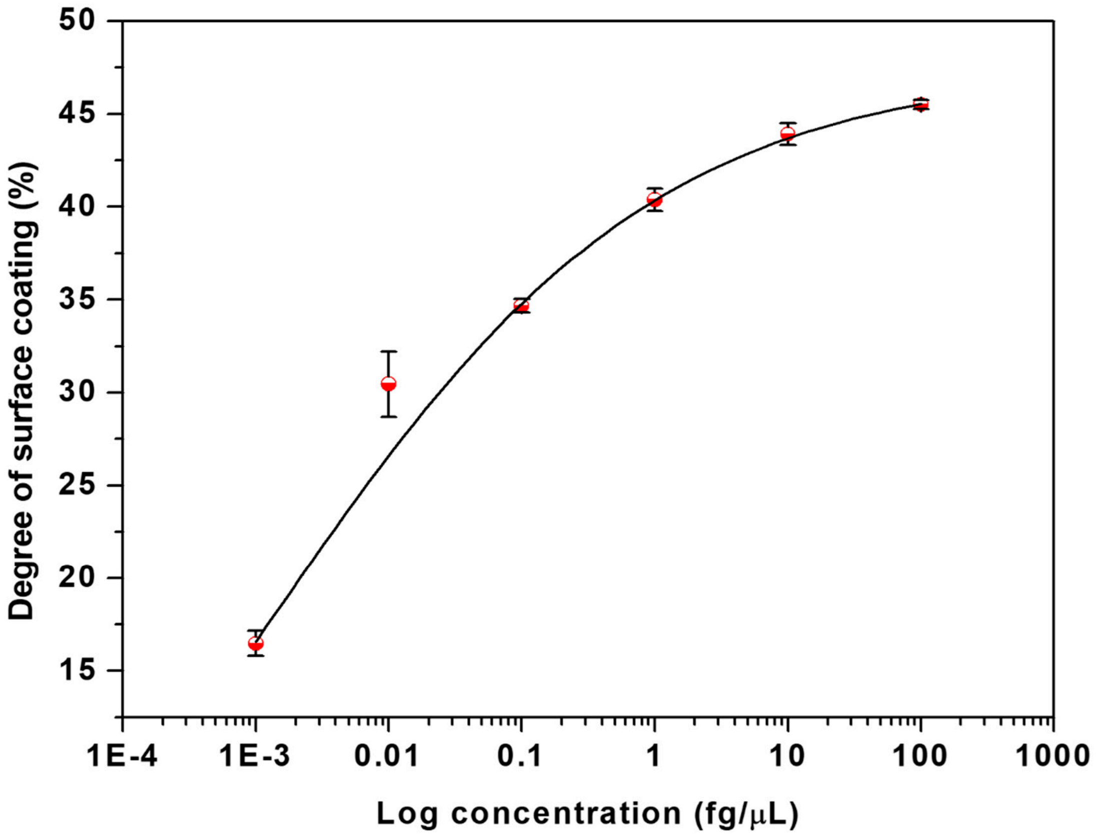

Table S1. Oligonucleotide sequences of primers and probes employed in the molecular biology assays.

	**Oligonucleotide sequence**
**Plasmid**	5'CACAGCCGGCGACATGCACACGCTGCTGCCTGGCCACGGGGCGCTGGCC TCAGGTTTCACCGGCCCCATGTCACTGGGCGGGCGGCACGCAGGCCTGGTT GGAGGCAGCCACCCCGAGGACGGCCTCGCAGGCAGCACCAGCCTCATGCA CAACCACGCGGCCCTCCCCAGCCAGCCAGGCACCCTCCCTGACCTGTCTCG GCCTCCCGACTCCTACAGTG|TTTTGAGTATCCGAGGAGCCCAGGAGGAAG AACCCACAGACCCCCAGCTGATGCGGCTGGACAACATGCTGTTAGCGGAA GGCGTGGCGGGGCCTGAGAAGGGCGGAGGGTCGGCGGCAGCGGCGGCAG CGGCGGCGGCTTCTGGAGGGGCAGGTTCA3'
**Conventional PCR primers**	
E2A2	5'CACAGCCGGCGACATGCACAC3'
PBX1-2	5'TGAACCTGCCCCTCCAGAAGC3'
**qPCR primers**	
ENF101	5'CCAGCCTCATGCACAACCA3'
ENP141	5'CCCTCCCTGACCTGTCTCGGCC3'
ENR161	5'GGGCTCCTCGGATACTCAAAA3'
**Sensing probe**	H_2N-5'CCCTCCCTGACCTGTCTCGGCC3'

Table S2. Anodic and cathodic peak currents during the construction steps of the DNA biosensor after exposure to analytical samples. Three consecutive analyses were conducted for each methodological procedure, and the experimental values are reported as the mean values ± standard deviation.

**Modified electrode**	**Sample concentration**	**Cathodic peak current (µA)**	**Anodic peak current (µA)**	**ΔI (%)**
Gold electrode	--	-76.24 ± 0.33	78.36 ± 0.16	--
PPy	--	-97.25 ± 2.75	106.30 ± 1.11	--
PPy-TiO_2@Pt	--	-64.45 ± 0.60	69.58 ± 1.98	--
PPy-TiO_2@Pt-Probe	--	-45.94 ± 0.43	48.75 ± 1.26	--
PPy-TiO_2@Pt-Probe-BSA	--	-46.12 ± 1.19	46.90 ± 0.45	--
Sensitivity assay (recombinant plasmid)				
Biosensor	--	-46.12 ± 1.19	46.90 ± 0.45	--
Biosensor-Oncogene t(1;19)	35.77 aM	-32.78 ± 1.47	46.43 ± 0.71	1.04 ± 1.55
Biosensor-Oncogene t(1;19)	357.67 aM	-29.23 ± 2.30	42.49 ± 0.26	10.39 ± 0.68
Biosensor-Oncogene t(1;19)	3.58 fM	-24.45 ± 1.73	40.16 ± 0.10	16.79 ± 0.28
Biosensor-Oncogene t(1;19)	35.77 fM	-23.47 ± 2.63	35.74 ± 0.11	31.24 ± 0.40
Biosensor-Oncogene t(1;19)	357.67 fM	-18.45 ± 3.00	29.64 ± 0.29	58.27 ± 1.57
Sensitivity assay (specimen from patient with ALL)				
Biosensor-Clinical Sample t(1;19)	1 ag/µL	-19.96 ± 0.83	35.24 ± 0.08	33.08 ± 0.28
Biosensor-Clinical Sample t(1;19)	10 ag/µL	-16.10 ± 0.99	26.06 ± 1.63	80.47 ± 11.64
Biosensor-Clinical Sample t(1;19)	100 ag/µL	-13.86 ± 0.61	23.26 ± 1.29	102.10 ± 11.40
Biosensor-Clinical Sample t(1;19)	1 fg/µL	-12.79 ± 0.44	22.32 ± 1.21	110.56 ± 11.65
Biosensor-Clinical Sample t(1;19)	10 fg/µL	-12.71 ± 0.58	21.67 ± 1.68	117.29 ± 17.35
Biosensor-Clinical Sample t(1;19)	100 fg/µL	-12.49 ± 0.39	20.93 ± 1.55	124.91 ± 17.08

Table S3. The values of the equivalent circuit elements were obtained by fitting the impedance results for each step of biodevice assembly. Three consecutive analyses were conducted for each methodological procedure, and the experimental values are reported as the mean values ± standard deviation.

**Modified electrode**	**Sample concentration**	**R_{CT} (kΩ)**	**ΔR_{CT} (%)**	**ϴ (%)**
Gold electrode	--	0.45 ± 0.004	--	--
PPy	--	-38.25 ± 1.48	--	--
PPy-TiO_2@Pt	--	2.28 ± 0.04	--	--
PPy-TiO_2@Pt-Probe	--	4.19 ± 0.07	--	--
PPy-TiO_2@Pt-Probe-BSA	--	4.21 ± 0.01	--	--
Sensitivity assay (recombinant plasmid)				
Biosensor	--	4.21 ± 0.01	--	--
Biosensor-Oncogene t(1;19)	3.58 aM	4.56 ± 0.11	8.10 ± 2.70	7.46 ± 2.33
Biosensor-Oncogene t(1;19)	35.77 aM	5.15 ± 0.04	22.18 ± 1.09	18.15 ± 0.73
Biosensor-Oncogene t(1;19)	357.67 aM	5.98 ± 0.01	41.95 ± 0.14	29.55 ± 0.07
Biosensor-Oncogene t(1;19)	3.58 fM	6.58 ± 0.02	56.11 ± 0.41	35.94 ± 0.17
Biosensor-Oncogene t(1;19)	35.77 fM	7.31 ± 0.17	73.35 ± 4.04	42.29 ± 1.35
Biosensor-Oncogene t(1;19)	357.67 fM	8.22 ± 0.37	95.14 ± 8.89	48.70 ± 2.34
Sensitivity assay (specimen from patient with ALL)				
Biosensor-Clinical Sample t(1;19)	1 ag/µL	5.05 ± 0.04	19.73 ± 0.96	16.48 ± 0.67
Biosensor-Clinical Sample t(1;19)	10 ag/µL	6.07 ± 0.20	43.93 ± 4.80	30.47 ± 2.36
Biosensor-Clinical Sample t(1;19)	100 ag/µL	6.45 ± 0.03	53.10 ± 0.83	34.68 ± 0.36
Biosensor-Clinical Sample t(1;19)	1 fg/µL	7.07 ± 0.07	67.73 ± 1.68	40.38 ± 0.60
Biosensor-Clinical Sample t(1;19)	10 fg/µL	7.52 ± 0.08	78.33 ± 1.84	43.92 ± 0.58
Biosensor-Clinical Sample t(1;19)	100 fg/µL	7.73 ± 0.03	83.51 ± 0.84	45.51 ± 0.25
Specificity assay				
Biosensor-Oncogene t(4;11)	355.19 fM	4.38 ± 0.09	3.91 ± 2.13	3.74 ± 1.98
Biosensor-Oncogene t(17;19)	374.62 fM	4.53 ± 0.08	7.39 ± 1.99	6.86 ± 1.74
Biosensor-Clinical Sample t(4;11)	100 fg/µL	4.21 ± 0.03	-0.12 ± 0.63	-0.12 ± 0.63
Biosensor-Clinical Sample t(17;19)	100 fg/µL	4.09 ± 0.08	-2.96 ± 1.94	-3.08 ± 2.08
Analytical interference assay				
Biosensor-Glycine	100 mg/dL	4.08 ± 0.04	-3.12 ± 0.90	-3.23 ± 0.96
Biosensor-Glucose	100 mg/dL	4.29 ± 0.08	1.86 ± 2.02	1.79 ± 1.94
Biosensor-Ascorbic Acid	100 mg/dL	4.09 ± 0.07	-2.89 ± 1.78	-2.99 ± 1.89
Biosensor-Cholesterol	100 mg/dL	4.26 ± 0.12	1.19 ± 2.85	1.13 ± 2.79

Reference

1. Wu, X.; Chai, Y.; Zhang, P.; Yuan, R., An electrochemical biosensor for sensitive detection of microRNA-155: combining target recycling with cascade catalysis for signal amplification. *ACS applied materials & interfaces* **2015,** 7, (1), 713-720.
